# 13 Plus 1: A 30-Year Perspective on Microtubule-Based Motility in *Dictyostelium*

**DOI:** 10.3390/cells9030528

**Published:** 2020-02-25

**Authors:** Michael P. Koonce

**Affiliations:** Division of Translational Medicine, Wadsworth Center, NYS Department of Health, Albany, NY 12201-0509, USA; michael.koonce@health.ny.gov; Tel.: +1-518-486-1490

**Keywords:** microtubules, dynein, kinesin, *Dictyostelium*, cell motility

## Abstract

Individual gene analyses of microtubule-based motor proteins in *Dictyostelium discoideum* have provided a rough draft of its machinery for cytoplasmic organization and division. This review collates their activities and looks forward to what is next. A comprehensive approach that considers the collective actions of motors, how they balance rates and directions, and how they integrate with the actin cytoskeleton will be necessary for a complete understanding of cellular dynamics.

## 1. Introduction

The microscopic interactions of motor proteins and the cytoskeleton underlie a number of important actions in eukaryotic cells, including organelle transport, cell shape change, and cell division. Nanometer scale steps act locally to produce order, tension, and directionality in the cytoplasm and yet often translate into macroscale movements that serve as the defining visual features of life. Large families of motor proteins have evolved to carry out multiple tasks, some with specific and unique functions, others that appear to overlap or provide redundancy. Identification of these families began in earnest in the 1970s and 1980s and matured with the widespread application of genome sequencing.

We now know exquisite detail about how some of the motors interact with their substrates and how they generate force, and are beginning to understand how they are regulated. Perhaps less understood is family diversity, how each motor isoform in a particular organism contributes to cellular function and the level of redundancy in their actions. 

This review focuses on the microtubule (MT)-based set of motors and in the compact organism *Dictyostelium discoideum*. It summarizes what we currently know about the collective actions of the 13 kinesins and one dynein and offers three directions for future study. *D. discoideum* is particularly useful here for a couple of reasons: it exhibits a robust level of intracellular motility, the number of motor isoforms affords diversity without being hugely complex, and is readily amenable to experimental characterization.

## 2. Microtubule-Based Motilities

A striking feature of the *D. discoideum* cytoplasm is the rapid and conspicuous movement of cellular organelles. Most of the visual motility is dependent on the MT cytoskeleton and was first carefully quantitated in [1]. In that study, rates of movement ranged from 1.4 to 2.8 μm/s and were dependent on particle size (smallest were the fastest). More recent quantitations using fluorescently tagged dynein markers [2] and in vitro reconstituted organelle assays [3,4] are consistent with this range. Not only do organelles move, but the interphase MT framework is also highly mobile, with many lateral bending motions of single MTs and of MT arcing along the inner surface of the cell cortex [5,6,7]. These movements occur at rates around 1 μm/s [8] and are likely due to the pushing/pulling actions of kinesin and dynein motors positioned on endomembranes or anchored at the cell cortex.

On mitotic entry, the cytoplasmic MT array rapidly disassembles, resulting in the abrupt cessation of organelle movements. The centrosome inserts into the nuclear envelope and a compact, rod-shaped intranuclear spindle forms between the two separating spindle poles. Astral MTs also project into the cytoplasm, though they are not as conspicuous as the central spindle. Spindles elongate during anaphase to about 10 μm in length, at rates from 1.6 to 4 μm/min [9,10]. 

Collectively, these cellular dynamics are powered by machinery interacting with the MT array. In the absence of MTs, organelle motility is nearly absent, spindles fail to form, and cells do not divide [1,11]. To support these movements, the motors must perform tasks that both affect individual actions and balance their collective activities. Each of the 14 MT-based motors in *D. discoideum* has been targeted for disruption. Table 1 provides a brief summary and the text below discusses these results.

## 3. Microtubule-Based Motor Machinery

### 3.1. Dynein 

Genome analyses demonstrate a single isoform of the minus-MT-end-directed cytoplasmic dynein in *D. discoideum*, and indicate that its deletion is lethal [2,12,13]. *D. discoideum* lacks cilia or flagella and thus does not contain genes for the axonemal family members. Although chemical inhibitors such as ciliobrevin or dynarrestin are non-effective in this organism, dynein function can be impacted through dominant negative expression of motor fragments [6,14], as well as manipulations of the DdLis1 and dynein intermediate chain [15,16] and the XMAP215 homologue DdCP224 [17]. In these cases, the interphase MT array uncouples from cortical anchorages and becomes motile in the cytoplasm, forming comet-like arrays. There are also significant impacts on minus-MT-end-directed organelle transport, organization of the Golgi, as well as centrosome duplication and spindle mechanics during cell division [4,15]. These studies indicate that dynein participates in both interphase and mitotic activities, with a range of actions commonly seen in other organisms.

### 3.2. Kinesins 

The first kinesin-like activity in *D. discoideum* was reported in 1989 [18], glimpses of the multigene family were revealed in 1998 [19], and the entire family of 13 isoforms was comprehensively presented in 2003 [20]. All members of this kinesin family have been individually targeted for disruption; some produce familiar phenotypes, others with novel or no obvious defects. Only two isoforms appear essential for viability, DdKif3 (Kinesin-1) and DdKif6 (Kinesin-13), and five isoforms show no obvious phenotypes during vegetative growth (Table 1). 

#### 3.2.1. Organelle Transporters (DdKif1, DdKif3) 

The DdKif1 kinesin (Kinesin-3) appears to be the primary motor to power plus-end-directed organelle transport in *D. discoideum*. There is a 62% reduction in overall organelle transport in Dd*Kif1* null cells (where MT polarity is not always obvious) and up to a 90% reduction in MT plus-end-directed activity using an in vitro reconstituted organelle assay [3]. Isolated organelles, stripped of motors and then incubated with purified DdKif1, moved with an average rate of 2.6 μm/s, a speed consistent with even the fastest organelles seen in *D. discoideum*. 

Attempted knockouts of Dd*Kif3* (Kinesin-1 family) indicate this motor is likely essential for viability [22,23], though in what capacity remains unknown. DdKif3 can be added to stripped vesicle populations and it induces plus-end-directed organelle movements (1.9 μm/s, [3]). This result indicates that there are appropriate receptors on the vesicles to dock the motor in a functional way, suggesting that it also participates in organelle transport. 

#### 3.2.2. Potential MT Connectors (DdKif5, DdKif7) 

A knockout of Dd*Kif5* (Kinesin-1 family) shows no effect on cell growth or development [24]. Interestingly though, the carboxy-terminal tail binds directly to actin and can bundle actin filaments in vitro. Antibody labeling of the protein colocalizes with actin-rich cellular protrusions and in the cleavage furrow, suggesting that this motor facilitates connections between MTs and the actin cytoskeleton.

DdKif11 is one of two isoforms of the Kinesin-7 family. A gene knockout does not reveal an obvious phenotypic defect [21,25], however, this protein localizes along MTs and is enriched near their plus ends, a site typically associated with MT dynamics, linkages, or cargo stability [31].

#### 3.2.3. Dynein Antagonists (DdKif8, DdKif10) 

Isoforms of the Kinesin-4 (DdKif8) and Kinesin-8 (DdKif10) families do not appear to play critical roles in cell growth, motility, or development in *D. discoideum* [21]. A knockout of Dd*Kif8* produces interphase MT arrays that appear longer and less radially organized in the cytoplasm, but no other differences are noted. Members of both kinesin families play prominent roles in MT length control, and in particular during mitotic activities in many animal cells [32,33,34,35,36,37]. However, there are no indications of mitotic function in *D. discoideum* and instead, these two motors may act in concert with dynein to organize and balance interphase MT organization. For example, we were unable to produce the distinctive comet-like motile MT behavior induced by dynein motor domain overexpression in either kinesin null cell background [21].

#### 3.2.4. Spindle Assembly (DdKif9, DdKif6, DdKif4)

DdKif9 is a novel Kin-I motor with a carboxy-terminal transmembrane domain that anchors it into the nuclear envelope [27,28]. It functions to help maintain close proximity of the centrosome to the nucleus to facilitate insertion into the nuclear envelope for mitosis. In the absence of centrosome-nuclear-envelope pairing, mitotic entry is stalled and leads to failed karyokinesis and supernumerary centrosome formation [38].

DdKif6 (Kinesin-13) and DdKif4 (Kinesin-7) provide the core kinesin activities to build the spindle properly and to connect chromosomes. In many animal cells, these two isoforms are required for managing MT dynamics during spindle assembly and for connecting kinetochores to spindle MTs [39,40,41]. Attempts to knockout Dd*Kif6* function in *D. discoideum* were unsuccessful, however, expression of an inducible RNAi hairpin targeting DdKif6 expression produces significant chromosome attachment and spindle organization defects [28]. These results are consistent with the phenotypes seen in other studies. 

Although the deletion of Dd*Kif4* is viable, null cells grow at significantly reduced rates [21]. Curiously, MT defects only become visible in Dd*Kif4* null cells when challenged by dynein perturbation, with obvious defective spindle arrangements.

#### 3.2.5. Spindle Elongation and Cleavage (DdKif13, DdKif12)

In most animal cells, Kinesin-5 participates in the spindle overlap region to organize and drive pole separation [42]. In *D. discoideum*, Dd*Kif13* (Kinesin-5) deletion reveals only subtle changes in spindle activity, a slightly elevated rate of elongation (1.9 vs. 1.6 μm/min) and after achieving maximal length, the two spindle halves prematurely separate [10]. 

Kinesin-6 molecules typically function in coordinating spindle activity with cleavage furrow formation and cytokinesis [43]. Defects in Dd*Kif12* (Kinesin-6) null cells are consistent with these functions, including malformed cleavage furrows, reduced rate of nuclear separation, and frequent metaphase arrest [29]. These cells are unable to grow in suspension and require a surface-assisted pathway for cellular fission (cytokinesis B, [44,45].

### 3.3. Developmentally Regulated 

*D. discoideum* has a vegetative growth stage where a single amoeba crawls, feeds, and divides. Starvation triggers a cAMP signaling cascade to aggregate cells into groups of about 10^5^ cells and initiates a developmental program to form spore-filled capsules lifted off the substrate on the top of stalks. DdKif2, DdKif7 (Kinesin-14 and Kinesin-1) do not appear to be expressed during vegetative growth but mRNAs are present after 8 h of starvation [19]. Gene knockouts of either motor do not reveal any significant vegetative cell defects [19,30]. 

## 4. Discussion

Of the 14 MT-based motor proteins in *D. discoideum*, only seven make clear and impactful individual contributions to cellular activities during vegetative growth. Dynein is obviously a major player in both interphase and mitotic activities, and only two of the kinesins are absolutely essential for viability. Four other motors make significant contributions, providing the organism with two dominant kinesins for plus-end organelle transport, two for spindle assembly, one motor for mitotic entry, and one motor for mitotic exit (cytokinesis). The seven other kinesins either make less impactful individual contributions to cell activity or they function in capacities outside of the conditions required for vegetative growth in a Petri dish. 

Some highlights to the motor analyses in *D. discoideum* include:

The two dominant MT-plus-end organelle transporters exhibit in vitro rates that are roughly 4–5 times faster than the traditional conventional kinesin (~2.5 vs. 0.5 μm/s). The in vitro work nicely complements the elevated rates of organelle motility seen in vivo, suggesting that *D. discoideum* would be an interesting model to investigate kinesin modifications that govern motor rates. It is intriguing that one isoform accounts for most of the observed plus-end-directed traffic, and yet is not the isoform required for viability. This issue deserves a second look, perhaps with an array of inducible inhibitors.

A couple of the motors appear evolutionarily tuned for the *D. discoideum* biology. *D. discoideum* is one of a few organisms known to have a centrosome positioned in the cytoplasm during interphase but can only promote spindle assembly within the nuclear compartment. DdKif9 has a unique function to keep the centrosome adjacent to the nucleus to aid its transition into the nuclear envelope for mitosis. Its anchorage at the nuclear envelope limits its cellular distribution and the adaptation of an internal motor arrangement likely fosters an MT-binding activity that draws the centrosome close to the nucleus [27].

Also unusual, Kinesin-5 is a motor essential for bipolar spindle function in most animal cells but is dispensable in *D. discoideum*. DdKif13 appears here in a support capacity, not so much to power but to guide spindle elongation. This function may reflect an evolutionary difference in spindle elongation mechanisms, where here and in some other organisms, astral pulling forces dominate over MT pushing for anaphase-B elongation [46]. The rapid pole separation seen in *D. discoideum* cells that lack a proper spindle overlap zone (e.g., [47]) suggests that cortical dynein pulling forces may be the primary driver for spindle elongation, and thus DdKif13 may be fine-tuned for a governor-type activity in the spindle midzone. In support of this idea, it is interesting to note that the primary antagonist of Kinesin-5 motors in animal-cell spindle assemblies is Kinesin-14; these two motors typically counterbalance actions to maintain spindle bipolarity [48,49]. Although *D. discoideum* does contain a Kinesin-14 isoform, this motor does not appear to be expressed in vegetatively-growing cells, and thus a robust Kinesin-5 pushing action may not be necessary during mitosis.

This collective rough draft of MT motors in *D. discoideum* further highlights the challenges even in compact organisms to identify functional detail, particularly with some *D. discoideum* motors that have well-characterized activities in other organisms (e.g., Kinesin-4, Kinesin-8, and Kinesin-14). To be fair, *D. discoideum* also undergoes a developmental program to form spores and a multifaceted sexual cycle where specialized motor activities may be relevant and disrupted and simply need to be viewed in the proper context.

Looking forward, the work leads into three interesting directions:

A. Developing a minimal motor paradigm.

Going forward, it should prove interesting to delete multiple kinesin isoforms in this organism and establish a minimal motor ensemble that maintains viability. The implementation of CRISPR/Cas9 gene-editing tools in *D. discoideum* [50] now makes this strategy feasible. The first screen ought to begin with the motors that show no obvious phenotypes or are not expressed in vegetatively-growing cells. Can one truly remove half of the MT-based motors and retain a viable cell, and under what growth conditions? The multiple cellular activities, the biochemical strategies, and amenable in vitro assays make *D. discoideum* an attractive model for such a minimalist approach. 

B. Understanding how transport rates affect cellular dynamics.

The two dominant MT-plus-end organelle transporters power movement significantly faster than the traditional conventional kinesin. It would be interesting to swap in motor isoforms from other organisms and compare functional properties. This could readily be performed as chimeras, coupling external motor domains with native cargo-binding regions. Can a mammalian conventional kinesin drive organelle transport in *D. discoideum*, and if so, does reducing the rate of motility have any impact on organelle distribution or cellular activity?

C. Providing a model to explore the integration of actin and MT cytoskeletons. 

*D. discoideum* also contains a robust actin filament system that is supported by 13 myosin motors [51]. There is surely crosstalk and integration between the two cytoskeletal systems that are important for a comprehensive understanding of cell motility. The DdKif5 (Kinesin-1) isoform that crosslinks actin in vitro stands out; on paper, this motor would appear to play a very interesting role in integrating the two filament systems, but in practice, there are few or no phenotypic consequences to its removal. The myosins—particularly those that contain MyTH4-FERM domains—are also likely to mediate crosstalk between the two filament systems [52,53]. How might one challenge cells to tease out functional components to this interaction? 

The individual analyses and deletions over the past thirty years have served well to provide a baseline for MT-based motor activities in *D. discoideum*, as have similar works in many other systems. The next steps ought to look larger at the collective acts of these motors and how these integrate their steps with each other. *D. discoideum* seems well-poised for this higher-ordered type of analysis but will require thoughtful and creative ways to poke and prod its motilities to glean a greater understanding.

## Figures and Tables

**Table 1:** A summary of the microtubule (MT)-based motor proteins in *Dictyostelium discoideum*. For detailed schematic representations of the kinesin motors organization, see references [20,21].

Dd Name	Family	In Vivo Rate	Primary Phenotype	Refs
DhcA	Cytoplasmic dynein	1.0 μm/s (motor domain)	Essential gene—interphase and mitotic functions	[12,13]
Kif1	Kinesin-3	2.6 μm/s	KO—90% decrease in MT plus-end-directed organelle motility	[3]
Kif3	Kinesin-1	2.0 μm/s	Essential gene—probably an organelle transporter	[22,23]
Kif5	Kinesin-1	nd	KO—no obvious phenotype, but motor binds to actin	[24]
Kif11	Kinesin-7	0.038 μm/s	KO—no obvious phenotype, motor localizes to cytoplasmic MTs, enriched near plus-ends	[21,25]
Kif8	Kinesin-4	1.6 μm/s	KO—MTs appear longer but no impact on growth or development	[21,26]
Kif10	Kinesin-8	0.17 μm/s (motor+ domain)	KO—no obvious phenotype	[21]
Kif9	Novel Kin-I	MT depolymerize	KO—promotes centrosome detachment from nucleus, impairs mitotic entry/spindle assembly	[27,28]
Kif6	Kinesin-13	nd	Essential gene—RNAi knockdowns reveal chromosome attachment and spindle defects	[28]
Kif4	Kinesin-7	nd	Slow growth in null cells—spindle/chromosome defects when coupled with dynein perturbation	[21]
Kif13	Kinesin-5	nd	KO—spindles prematurely slide apart during anaphase	[10]
Kif12	Kinesin-6	nd	KO—cells unable to complete cytokinesis in suspension	[29]
Kif2	Kinesin-14	nd	KO—no vegetative cell defects. Developmentally regulated	[19]
Kif7	Kinesin-1	0.14 μm/s (motor+ domain)	KO—no vegetative cell defects. Developmentally regulated	[30]
